# Diet in the Typhoid State

**Published:** 1899-06-24

**Authors:** 


### DIET IN THE TYPHOID STATE.

Why is the tongue dry in the "typhoid state?" meaning thereby not typhoid fever alone, but that advanced condition of prostration which, although found in a typical form in the later stages of enteric fever, is also met with in typhus fever, pneumonia, and pyæmia, and often towards the close of phthisis and many other diseases accompanied with fever, exhaustion, and cardiac failure. Dr. Dickinson says that in this condition the tongue is dry because no saliva is secreted, and that there is much reason to believe that what happens to the salivary glands in these cases happens also to other glandular structures concerned in digestion, so that a dry tongue may be taken as a sure sign that, as well as the accompanying loss of appetite, there is also a loss of digestive power. What has then to be done under such circumstances is to give only liquids—that is to say, foods on which one part of the digestive process, and that probably the most difficult part, has been accomplished. Dissolved food can be absorbed and utilised with the minimum of digestive effort. Milk is a complete nutriment. Eggs in their entirety—that is, yolks and whites together—may also be regarded as a complete nutriment. Beef-tea takes the next place to milk in the accepted dietary of the typhoid state. It contains the animal extracts, such as are found in Liebig's Extract, together with gelatin and (in the sediment) albumin. But it contains only a trace of fat, and thus is wanting in carbonaceous materials. It must, however, be remembered that even the most nitrogenous components of our food contain three times as much carbon as nitrogen, and that, as we see in diabetes, a considerable amount of hydrocarbon may be set free even out of a diet which consists as strictly as possible of foods of the nitrogenous class. Still it is better, where the digestive power is so enfeebled as in the cases under consideration, to add the essential carbon ready for use in a liquid form, and for this purpose sugar supplies exactly what beef-tea is wanting in. Arrowroot, which itself is almost pure starch, is also a convenient vehicle for sugar. Both milk and beef-tea may be peptonised in extreme conditions, but generally if the food is presented in a liquid form the stomach may be left to do its own work in regard to absorption. Passing now from the typhoid state in general to typhoid fever in particular, the necessity of the addition of sugar and starch must be insisted on whenever the diet consists largely of beaf-tea. Brandy and egg mixture must also be remembered as an important means of completing the diet in the typhoid state, whether this occurs in the course of typhoid fever or not.

